# Topological constraints on network control profiles

**DOI:** 10.1038/srep18693

**Published:** 2015-12-22

**Authors:** Colin Campbell, Justin Ruths, Derek Ruths, Katriona Shea, Réka Albert

**Affiliations:** 1 Department of Physics, Pennsylvania State University, 104 Davey Laboratory, University Park, PA 16802; 2 Department of Biology, Pennsylvania State University, 208 Mueller Laboratory, University Park, PA 16802; 3 Department of Physics, Washington College, 300 Washington Avenue, Chestertown, MD 21620; 4 Engineering Systems and Design, Singapore University of Technology and Design, 8 Somapah Road, Singapore 487372; 5 Department of Computer Science, McGill University, McConnell Engineering Bldg. Room 318, 3480 University, Montreal, Qc, H3A 0E9, Canada

## Abstract

Network models are designed to capture properties of empirical networks and thereby provide insight into the processes that underlie the formation of complex systems. As new information concerning network structure becomes available, it becomes possible to design models that more fully capture the properties of empirical networks. A recent advance in our understanding of network structure is the control profile, which summarizes the structural controllability of a network in terms of source nodes, external dilations, and internal dilations. Here, we consider the topological properties–and their formation mechanisms—that constrain the control profile. We consider five representative empirical categories of internal-dilation dominated networks, and show that the number of source and sink nodes, the form of the in- and out-degree distributions, and local complexity (e.g., cycles) shape the control profile. We evaluate network models that are sufficient to produce realistic control profiles, and conclude that holistic network models should similarly consider these properties.

Complexity is an emergent property of a diverse array of biological^1^,^2^,^3^,^4^, technological^5^,^6^,^7^, and social^8^,^9^,^10^,^11^ systems. As such, the controllability of complex systems is a topic of intense theoretical and applied interest^12^,^13^,^14^,^15^,^16^. In the context of *structural controllability*, complex systems may be abstracted into networks that consist of nodes (system components) and unweighted edges that connect pairs of nodes (interactions and relationships among components). The system components are often assumed to omit self-regulation (cycles of length 1) and obey linear dynamics of the form


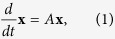


where **x** is a vector of length *N* (the number of nodes in the network) and *A* is a matrix that quantifies node-node influence (i.e., network edges). The broad objective of network control is to determine a minimal modification to the network that confers the ability to drive the entire system from any dynamic state to any other dynamic state, for instance by feeding *N*_*c*_ independent signals (controls) into a subset of 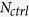 “controlled” nodes (where 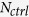 may be larger than *N*_*c*_ due to the presence of certain types of cycles) such that their dynamics instead obey


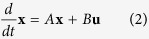


where **u** is a vector of the time-varying control signals and *B* has 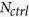 nonzero entries indicating where they are fed into the network. Existing work has found that the value of *N*_*c*_ is dependent on the network's degree distribution^12^. More specifically, the density of nodes with a low in-degree and/or out-degree strongly influences the controllability of a network^15^, and in many cases the value of *N*_*c*_ is largely determined by the relative abundance of source and sink nodes^13^, which we here denote respectively as *N*_*src*_ and *N*_*snk*_. Moreover, Ruths and Ruths^13^ categorized the types of controls into those caused by *source nodes*, *external dilations*, and *internal dilations*. The number of controls in each category is respectively defined as:


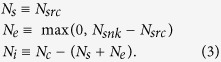


*N*_*src*_ and *N*_*snk*_ may be directly calculated in 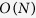 time, and the most common algorithm for calculating *N*_*c*_ is of 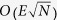 time for a network with *E* edges^13^; these quantities are therefore accessible for even very large networks. The ordered triple (*η*_*s*_, *η*_*e*_, *η*_*i*_) is defined by additionally normalizing these quantities by *N*_*c*_, and facilitates the comparison of the distribution of control types across networks of variable size and topology. This so-called *network control profile* reveals that empirical networks are generally dominated by one type of control^13^.

In their standard formulations, however, most common network models (probabilistically) create networks that are source dominated; i.e., 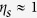
13. Therefore, new or modified models are needed that generate networks with control profiles dominated by internal or external dilations, as seen in real networks, while maintaining other key network properties. We have previously shown^17^,^18^ that the control profile (*η*_*s*_, *η*_*e*_, *η*_*i*_) of some network models may be tuned to some extent by modifying edge orientation. Models that generate networks through the iterative addition of nodes generally assign directed edges from the new node to pre-existing nodes^19^. In cases where the degree distribution is broad-tailed, the topology is accordingly *η*_*s*_-dominated. However, by simply reversing the directionality of all edges as nodes are added to the network, the abundance of source nodes becomes an abundance of sink nodes, and the network becomes *η*_*e*_*-*dominated^17^.

This is an intuitive approach that maps well to the empirical trends reported by Ruths and Ruths^13^. For instance, new participants in social settings are more likely to identify pre-existing participants as popular (*η*_*s*_ dominance), while social influence is more likely to be exerted in the opposite direction (*η*_*e*_ dominance). Randomizing the directionality of new edges, leading to a mixture of forward and reversed edges, directs the network control profile toward *η*_*i*_dominance; however, the appropriateness of this strategy in modeling real systems is unclear^17^,^18^.

Here, we systematically address this methodological gap by determining the key features required of network models to replicate the network control profiles of empirical networks. While we consider a wide range of empirical networks, we focus specifically on five classes of *η*_*i*_-dominated networks: airport-flight networks, the Internet at the level of autonomous systems, food webs, electronic circuits, and the World Wide Web. These network classes span natural (food webs), fabricated (electronic circuits), and dynamic technological networks with some (airports, the Internet) or minimal (WWW) centralized management. They are also topologically diverse, and are therefore likely to be representative of other classes of internally-dominated networks.

We show that there is no single mechanism that accounts for the existence of internal dilations across these empirical networks (in contrast to other control-related parameters, such as the number of controls, which is determined largely by the degree distribution^12^). While in some cases simple considerations of the network topology that arise from the empirical context constrain the network control profile to the desired subregion of the control profile space, in other cases the control profile is indicative of rich topological structures. Specifically, we show that in networks with broad degree distributions, the degree distributions are sufficient constraints on the control profile: randomizing network topology while preserving the degree distributions preserves the control profile. In cases where the degree distributions are not broad, the local topology dominates, and it is necessary to control for both the number of source and sink nodes and the nature of local interactions to preserve the control profile. Where applicable, we discuss viable generative models that create networks with the specified control profiles. In addition to offering insight into the mechanisms that give rise to internal dilations in empirical networks, these findings have direct implications for our understanding of the emergent properties of these complex systems and, ultimately, for our ability to effectively influence their behavior.

## Results

**Figure 1:**
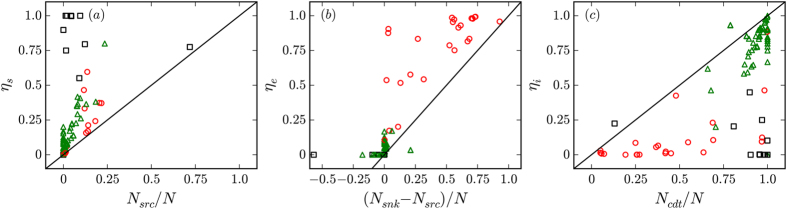
Relationships between the distribution of source (*N*_*src*_), sink (*N*_*snk*_) and conduit (*N*_*cdt*_) nodes and the network control profile parameters among 98 empirical networks. Networks with maximal control profile parameters of *η*_*s*_, *η*_*e*_, and *η*_*i*_ are respectively drawn with black squares, red circles, and green triangles. Black lines are drawn through the origin with a slope of 1 as a visual reference. (**a**) Each source node must be directly controlled, but networks with the largest values of *η*_*s*_ are not necessarily those with the largest relative fraction of source nodes. (**b**) In cases where the number of sink nodes greatly outweighs the number of source nodes, *η*_*e*_ is unambiguously the dominant control profile parameter; while sufficient, this is not necessary for a network to have a high value of *η*_*e*_. (**c**) Networks with high values of *η*_*i*_ are generally dominated by conduit nodes, but the converse does not hold.

We first consider the overall relationships between the relative abundances of sink, source, and conduit nodes (i.e., those that are neither source nodes nor sink nodes; *N*_*cdt*_ = *N* – *N*_*src*_ – *N*_*snk*_) and the control profiles for 98 empirical networks (see Supplementary Material for details). While some networks show a distribution between source nodes, sink nodes, and conduit nodes that is suggestive of a simple dependence of the control profile on the distribution of node types, there are many exceptions (Fig. 1). For example, networks whose control profiles are dominated by *η*_*s*_ generally have few source nodes (Fig. 1a), some networks with relatively low values of *(N*_*snk*_ − *N*_*src*_*)/N* nonetheless have high values of *η*_*e*_ (Fig. 1b), and some networks with high values of *N*_*cdt*_*/N* nonetheless have low values of *η*_*i*_ (Fig. 1c). Thus, in general, the control profile of empirical networks cannot be predicted from an assessment of the distribution of node types. We find similar behavior when considering the relationship between network control profiles and edge types or network density (Fig. S1).

Of particular interest is the relationship between a network’s degree distribution and its control profile. To investigate this, we consider five null models that preserve: (a) the number of nodes and edges, (b) the number of inputs and outputs, (c) the out-degree distribution (the out-degree of every node in the original network is uniquely assigned to a node in the randomized network), (d) the in-degree and out-degree distributions (the in-degree and out-degree are both randomized as in (c), independently from one another), and (e) the joint-degree distribution (the in-degree and out-degree of every node in the original network is uniquely assigned to a node in the randomized network).

**Figure 2:**
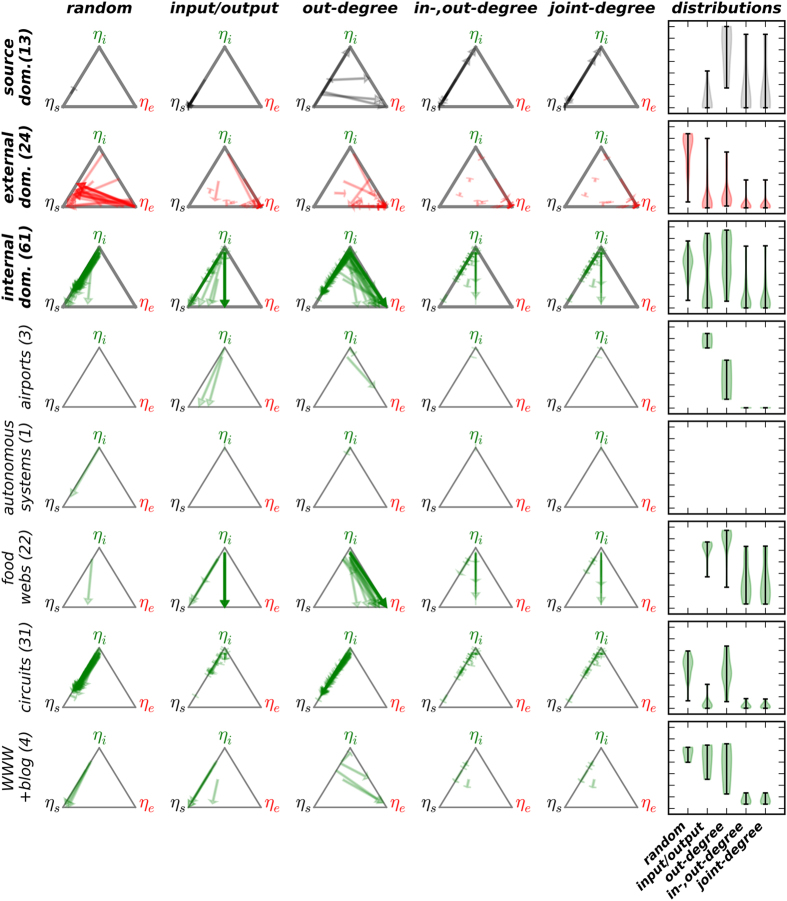
Behavior of the control profile of empirical networks under null models that preserve: (random) only the number of nodes and edges, (input/output) the number of source and sink nodes, (out-degree) the out-degree distribution and number of source and sink nodes, (in-,out-degree), both the in- and out-degree distributions, and (joint-degree) the joint-degree distribution. A total of 98 networks are categorized as dominated by *η*_*s*_, *η*_*e*_, or *η*_*i*_ (top three rows, respectively shown with black, red, and green vectors); the *η*_*i*_-dominated networks are further shown in five subcategories (bottom rows). In each panel, ternary plots show semi-transparent vectors pointing from the original control profile to the mean control profile over 10 randomizations (the low replication number is justified because the standard deviations are already very small at this point: mean <0.025, median <0.002). Vector tips are drawn with uniform width and length proportional to the overall length of the vector; as such, very short vectors are indicated with a thin line perpendicular to the orientation of the vector. Networks with a control profile = (0,0,0) are not shown^13^. The rightmost column shows the distribution of vector lengths (i.e., Cartesian distances) with a uniform vertical scale for each row, omitting plots with 0 or 1 vectors. The shorter the vector lengths, the better is the agreement between a null model and the empirical networks.

The salient question when considering each null model is how effective it is at preserving a network's control profile. We summarize the effect of each null model on the networks in Fig. 2. We find that control profiles whose dominant control profile parameter is either *η*_*s*_ or *η*_*e*_ are largely preserved by the conservative choice of simply controlling for the number of sources and sinks (as represented by short vector lengths). This is not the case, however, for networks whose dominant control profile parameter is *η*_*i*_; indeed, the behavior of these networks is varied and characteristic of empirical context. To identify the empirical network properties that constrain the control profile response to these null models, we consider several representative categories of these networks on a case-by-case basis.

### Airports

Airports by their nature both receive and dispatch aircraft. In network terminology this means that, in principle, airport nodes are neither sources nor sinks. This is indeed the case in the network comprising the connections among the 500 busiest airports in the world^20^. Accordingly, all null models that preserve the number of source nodes and sink nodes preserve the control profile of this network at *η*_*i*_ = 1.While other airport networks studied in the literature do have some source and sink nodes, they are rare, and their existence may be attributed to the methodology by which the networks are constructed. For instance, the network of all flights originating and/or terminating in a U.S. city include some international airports that only receive flights from, or dispatch flights to, the U.S., and some databases that have an international scope are not exhaustive^21^. However, even in these cases the total fraction of source and sink nodes is low (11% and 1%, respectively for the U.S. and international flight networks), and the vast majority of airport-airport interactions in these networks are bidirectional: if airport *A* receives flights from airport *B*, airport *B* typically receives flights from airport *A* (78%, 97%, and 91% of edges are paired in this way, respectively for the U.S., international, and 500 busiest airport networks).

Due to the presense of an appreciable number of source nodes in the U.S. and international airport networks, the control profile shifts to *η*_*s*_ dominance in the case of an input/output shuffle. In contrast, we find that the control profile of these networks is well-preserved when controlling for both the in- and out-degree distributions (Fig. 2). We further consider the degree distribution of the three above-mentioned empirical airport networks (Fig. S2). Because most interactions are bidirectional, the form of the in-degree distribution is similar to that of the out-degree distribution. Both the U.S. and international airport network in-degree and out-degree distributions obey truncated power laws, while, unsurprisingly, the size-restricted network of the 500 busiest airports in the world obeys a scaling law only for a small portion of its tail. For sufficiently large airport networks, then, a model should be constrained, at minimum, to (a) involve predominantly or entirely bidirectional edges (to generate desired control profiles) and (b) obey a truncated power law in its in- and out-degree distributions (to mimic the empirical degree distributions).

### The Internet

The Internet comprises interconnected routers, which may be simplified by considering connected sub-graphs referred to as Autonomous Systems^22^. While we consider specifically the network of Autonomous Systems of Ruths and Ruths^13^ and note that its in- and out-degree distributions are well-fit by log-normal, power law, or truncated power law distributions (Fig. S3), communication between Autonomous Systems over any appreciable period of time is inherently bidirectional. As such, these systems lack source and sink nodes and, therefore, *η*_*i*_ *=* 1. This intuitive finding is supported by the behavior of the control profile under network randomization; all randomization schemes that preserve the number of source and sink nodes preserve the control profile (Fig. 2). Models of the Internet will return the appropriate control profile if this feature is taken into consideration.

### Food Webs

Networks representing food webs comprise nodes that symbolize species and interactions corresponding to predation and energy flow through the web (i.e., A− > B indicates that members of species B consume members of species A). The study of food webs is an active area of ecological research^23^,^24^,^25^, as understanding their form and function is a necessary component of our broader understanding of ecological processes and stability. A food web may in general be closed (no sources or sinks) or open. We here consider the latter case; as above, the former would clearly return control profiles with *η*_*i*_ *=* 1. Empirical food webs are often too small to facilitate meaningful analysis of the form of the degree distribution, and indeed, the approaches to network randomization considered here generally fail to preserve the network’s control profile (Fig. 2). We note that while few interactions are bidirectional in these food webs (mean network value = 10%, considering 22 networks with over 15000 interactions), most species exist on at least one cycle (mean network value = 75%, considering 22 networks with over 1300 nodes). To simulate networks with realistic internal structures, we consider the niche model of Williams and Martinez^26^, which, despite its simplicity, replicates many important features of food webs (see Methods). In particular, we are interested in the ability of this model to duplicate the internally dominated control profiles of food webs.

We find that while food webs generated under this model often have large values of *η*_*i*_, they also can have nontrivial and often dominant values of *η*_*s*_, which can result in control profiles significantly different from those reported for empirical food webs (Fig. S4a). This is largely attributable to the relative abundance of source nodes (i.e., basal species with no prey) in networks generated by the niche model compared to the empirical food webs considered in Ruths and Ruths^13^. This in turn may be attributed to differing sampling procedures: some studies consider predation among large fish and disregard predation at lower trophic levels. By scaling the niche width according to niche value, the niche model tends to generate basal species at the low end of the niche-value unit interval.

A simple approach to address this discrepancy is to control for the number of source nodes in the network by rejecting surplus source species and repeating the creation process until the correct distribution is found. While a somewhat artificial approach, this forces the simulated food web to contain the empirically expected number of source nodes (i.e., producer species) while preserving the cannibalism and cyclic structure characteristic of the model. Taking this approach results in control profiles that closely match the empirical expectations (Fig. S4b). Thus, a combination of controlling for the internal complexity of food webs (via the niche model) and the relative abundance of source nodes yields realistic control profiles in simulated networks of food webs. We note that this approach will modify the density of the resulting networks. In a full treatment of such a modification to this model, this may be addressed by appropriately rescaling the model, though the implications of any such modification must be carefully considered in the context of the empirical food webs under consideration.

### Electronic Circuits

The ISCAS89 benchmark collection of 31 electronic circuits is defined by input nodes (sources), output nodes (sinks), and logical gates that route the inputs to the outputs^27^. Electronic circuits are a somewhat unique example of networks in that they are designed in their entirety before implementation and are generally not modified post-implementation. In contrast, other technological networks such as power grids, airports, and road networks are continually modified to meet changing societal demands. Thus, a dynamic growth model that mimics the structure of electronic circuits has limited utility, insofar as actual electronic circuits do not undergo a parallel growth process. We are, however, interested in the topological properties of these networks that gives rise to the *η*_*i*_dominance of their control profiles.

The number of input and output nodes grows slowly with network size (Fig. S5a) and the control profile becomes dominated by *η*_*i*_for large circuits (Fig. S5b). Indeed, there is an approximately linear relationship between the value of *η*_*i*_and the relative abundance of conduit nodes in the network (Fig. S5c). The maximal in-degree observed in these networks is 4; on average, 41% of a circuit's nodes have an in-degree of one and 42% have an in-degree of two. In contrast, on average 76% of a circuit's nodes have an out-degree of one; while the out-degree distributions span a broad range of values and decay faster than a power law, their form is not uniform (Fig. S6). Many nodes in these circuits, therefore, have a low in-degree and low out-degree. Some nodes, however, pair a low in-degree with a comparatively high out-degree^28^.

While the existence of such nodes can constrain the control profile to *η*_*i*_-dominance (see Discussion), we find that the simpler constraint of controlling the number of source nodes and sink nodes suffices to preserve the internal dominance of the control profile (Fig. 2). This is in contrast to the other networks with *η*_*i*_-dominated control profiles that contain both source and sink nodes (i.e., excluding the top 500 airport network and the autonomous systems network), and is likely due to the comparatively low connectivity in electronic circuits: the mean and median average degree across all electronic circuits is respectively 3.5 and 3.4, while for the other relevant networks with *η*_*i*_-dominated control profiles, these values are respectively 19.3 and 17.6.

### The World Wide Web

The World Wide Web (WWW) comprises web pages and the directed hyperlinks that join them. The in-degree and out-degree distributions for the WWW are well-approximated by power laws, though their exponents differ (Fig. S7a-d)^19^. The existence of nodes with high out-degree and low in-degree is therefore not as clear as in the case of electronic circuits. However, the WWW does contain such nodes, as evidenced by a consideration of the distribution of node degree differences (Fig. S7e-f). Preserving the in- and out-degree distributions under network randomization preserves the existence of these "low in, high out" nodes, a key topological property that both restricts the effective routing of control signals originating at source nodes and forms many internal dilations; both properties facilitate *η*_*i*_ dominance (see Discussion). Indeed, the *η*_*i*_ dominance of the WWW network control profiles are preserved under this randomization scheme (Fig. 2).

We independently verify this result by means of the configuration model, which, while not generative in the sense of dynamically growing a network, creates networks with precise degree distributions^29^. For each WWW network, we compare the average control profile of 10 networks generated via an implementation of the configuration model that preserves the joint-degree distribution to the average control profile of 10 randomized networks wherein the joint-degree distribution is maintained (see Methods). We then repeat this analysis where the in-degree and out-degree distributions (but not the joint-degree distribution) are maintained in both the generated and randomized networks. We find that 10 replications suffice in each case because the control profile parameter standard deviations are already quite small at this point (median <0.01 in all cases). As expected, we find close overlap between the control profiles according to these methods, although the joint-degree preserving approach offers stronger overlap (mean Cartesian distance = 0.03, median = 0.01 vs. mean = 0.29, median = 0.28).

## Discussion

The study of complexity has yielded significant insight into the structure and dynamics of complex systems from widely disparate fields of study^1^,^2^,^3^,^4^,^5^,^6^,^7^,^8^,^9^,^10^,^11^. A common methodological approach when probing the properties of complex networks is the development of models that replicate the key properties of empirical systems; preferential node attachment in growth models, for instance, leads to a degree distribution that obeys a power law, as is observed in many real networks^30^. In such a way we gain insight into the mechanisms at play in empirical networks.

Such an understanding informs our efforts to effectively influence the behavior of these complex systems; indeed, there is a growing body of literature concerning network controllability^12^,^13^,^14^,^15^,^16^. However, while our understanding of the structure and dynamics of complex networks informs our study of network controllability, the converse also holds—understanding control-related network properties informs our understanding of their structure and function. In particular, properties related to network control should be considered when developing holistic network models.

Here, we consider structural controllability in the sense of the control profile introduced by Ruths and Ruths^13^. In dynamic networks that obey equation (1), the control profile identifies the fraction of controls required (so that the entire network can be driven to any dynamic state) due to sources, sinks, and internal dilations (respectively denoted as *η*_*s*_, *η*_*e*_, and *η*_*i*_). We note that when preserving the number of source nodes and sink nodes, convergence in the control profile corresponds to convergence in the number of controlled nodes, as well.

The control profile is dominated by *η*_*s*_when (a) there is an abundance of source nodes or (b) the internal structure of the network facilitates complete or near-complete coverage by source control signals. In contrast, the control profile is dominated by *η*_*e*_when (a) there is an overabundance of sink nodes beyond source nodes (*N*_*e*_ > *N*_*s*_) and (b) the internal structure admits few internal dilations. When none of the above conditions are met, the control profile is dominated by *η*_*i*_.

Existing synthetic models generally return networks dominated by *η*_*s*_ due to an overabundance of source nodes, but in some cases these may be tuned to instead offer *η*_*e*_-dominance by reorienting edge directionality^17^,^18^. The main contribution of this work is the identification of the different classes of structures that relate to *η*_*i*_-dominance, i.e., the conditions where most nodes are neither sources nor sinks and the internal structure is complex enough to prevent the source control signals from reaching a vast majority of the network's nodes. More broadly, we identify three topological components that constrain the control profile: the in- and out-degree distributions constrain the control profile when the distributions are broad, while in cases where the degree distributions are not broad, the control profile is constrained by a joint consideration of the number of source and sink nodes and the local complexity of the network.

Notably, the properties that lead to the existence of internal dilations (and thereby *η*_*i*_ dominance of the control profile) are more complicated than the conditions that lead to *η*_*s*_ or *η*_*e*_ dominance. However, a simple model elucidates the role of these properties in constraining the control profile. Consider a hierarchical network composed of *m* layers where each layer contains *n* nodes and every node in layer *i* has an outgoing edge to every node in layer *i* + 1. Clearly, each of the *n* control signals on layer 1 may propagate to any of the *n* nodes on layer 2, then to any of the *n* nodes on layer 3, etc. Thus, *η*_*s*_ = 1 regardless of the number of layers (i.e., even in the limit *m* → ∞).

Directly from equation (3), increasing the number of sink nodes decreases *η*_*s*_ and increases *η*_*e*_. Deviations from this hierarchical structure while holding the number of source nodes and sink nodes constant corresponds to a decrease in *η*_*s*_ and an increase in *η*_*i*_; they may be considered in two categories. First, the control signals may not be able to propagate freely between adjacent layers. For instance, the number of nodes in adjacent internal layers may differ (corresponding to nodes with an out-degree larger than its in-degree or vice versa). In this case either some source control signals are restricted from propagating from layer *i* to layer *i* + 2 because the number of nodes on layer *i* is greater than the number of nodes on layer *i* + 1, or the source control signals are not sufficient to control all nodes on layer *i* + 1 because it contains more nodes than layer *i*. Even when the number of nodes on adjacent layers is identical, heterogeneity in connectivity between layers can provide the same restrictive effect. Second, the structure itself may not be hierarchical; real networks generally contain overlapping cycles that prohibit an unambiguous distribution of source control signals.

Real networks are neither hierarchical nor acyclic; the networks considered here highlight the roles of both of these properties in constraining the control profile. For instance, the degree distributions of food webs are generally narrow and fail to obey a well-defined scaling law. In this case, a simple model that captures both the appropriate number of source nodes and the form of food webs’ complex cyclic structure produces realistic (internally dominated) control profiles. In some cases the cyclic behavior is so extreme as to eliminate source and sink nodes altogether (e.g., autonomous systems), which directly leads to *η*_*i*_ = 1.

In contrast, networks with source and sink nodes and a broad degree distribution preserve their control profiles under null models that preserve their degree distributions (Fig. 2). The electronic circuits considered here have a narrow and low in-degree distribution (with a maximal value of 4) and a broad out-degree distribution, while the World Wide Web has broad in-degree and out-degree distributions. Both network types have nodes with a significantly greater out-degree than in-degree. In both cases, randomizing the degree distributions preserves this property and the *η*_*i*_-dominated control profile: in the case of electronic circuits, high out-degree nodes will necessarily retain low in-degrees, and in the case of the WWW, the few nodes with a high out-degree will probabilistically be assigned one of the abundant low in-degree values.

While this is a necessary constraint in the case of the WWW, the control profiles of electronic circuits are also preserved by the simpler null model that preserves only the number of source nodes and sink nodes (Fig. 2). This is likely due at least in part to the low mean degree in these networks. Indeed, randomizing the internal structure of a network that has many edges is likely to create many source-sink paths, and thereby produces networks with a *η*_*s*_-dominated control profile (unless *n*_*snk*_ ≫ *n*_*src*_, in which case the control profile would be dominated by *η*_*e*_). In contrast, a network with relatively few edges is likely to have few such paths, and *η*_*i*_-dominance follows. While models that preserve a network’s degree distributions, such as the configuration model^29^, broadly preserve a network’s control profile, in general it is clear that a holistic consideration of the relevant topological properties is necessary to completely capture a network’s control profile. For instance, the *s38584* circuit, which contains over 20,000 nodes, has a control profile of (*η*_*s*_, *η*_*e*_, *η*_*i*_) = (0.006, 0.045, 0.947). By preserving its joint-degree distribution through randomization, the mean control profile values are (*η*_*s*_, *η*_*e*_, *η*_*i*_) = (0.009, 0.060, 0.931) with standard deviations uniformly <0.001. The randomization therefore preserves the *η*_*i*_-dominance of the control profile, although the population means do not overlap with the profile of the original network (one-sample t test, *p* < 10^−5^ in all cases). Other properties that are destroyed via this randomization routine (e.g. local cyclic structures), clearly modulate the control profile even when the degree distributions are broad.

The degree distribution is a well-studied component of network topology, and many approaches exist to model empirically observed distributions. For instance, power law distributions are well-fit via preferential attachment based on node degree^30^; more flexibility in the fit is possible by including, for instance, a parameter corresponding to the initial node attractiveness^31^. More rapid decay, which is frequently observed in real systems, may be achieved by the inclusion of aging or limited node capacities^32^. In cases where the in-degree distribution is to be uncoupled from the out-degree distribution, such as the WWW, the inclusion of effects such as link addition after node addition may be applied^19^.

## Conclusions

One measure of our understanding of complex systems is the extent to which we can control their behavior. A crucial component of such an understanding is the identification of the salient control-related features of a complex system. Structural controllability theory offers a way to identify nodes to be controlled based on the interaction network alone. It is now known that the number of nodes that must be directly controlled to confer structural controllability is determined in large part by the network’s degree distribution^12^, and that these controlled nodes can be meaningfully classified according to their position in the network’s topology (e.g., as source, sink, or internal nodes)^13^. A natural next step is the identification of the context-specific mechanisms that give rise to this so-called *control profile*. Here, we have identified the in-degree distribution, out-degree distribution, and local structure (e.g., the existence of cycles) as the primary topological structures that constrain the control profile.

The task of integrating local interaction patterns with the degree distribution is comparatively unexplored in network modeling. More generally, greater characterization of the role of local interaction patterns (e.g. modularity, clustering, and motif structure) in structuring the internal complexity discussed here presents an intriguing topic for future work. An important consideration in this context is the role of other node and edge classification schemes that have been developed in the context of control theory. For instance, edges may be categorized based on the effect of their removal on the value of *N*_*ctrl*_^12^, and nodes may be categorized based on the frequency with which they exist as controlled nodes in alternative configurations of controlled nodes for a particular network^33^. Cycles were also identified as important control elements in systems obeying dissipative nonlinear dynamics^34^,^35^. Characterizing the topological aspects of controllability through a holistic analysis of such measures will facilitate deeper understanding of the interplay between the structural properties and dynamic behavior of controllable networks. In a broader context, extending the control profile (e.g., to consider dissipative nonlinear dynamics^34^,^35^, weighted interactions with self-loops^36^, or the interplay between network structure and the form of nodal dynamics^37^) and comparing its properties to the structural control profile, as considered here, stands to inform our understanding of the unique contributions of nonlinearity in control design.

## Methods

### Network Randomization

To randomize our interaction networks while preserving the degree distribution, we adapt the Curveball algorithm of Strona *et al.*^38^, a computationally efficient algorithm for randomizing matrices of binary elements. The algorithm is essentially an efficient implementation of the common edge-swap routine, although it makes important improvements concerning sampling bias^38^. The algorithm comprises the following steps: (a) select two unique nodes *α* and *β*; (b) identify the targets of *α* that are not targets of *β* and vice versa; (c) select *α'* and *β'*, nodes drawn from the sets identified in step (b), and interchange them such that *α* − > *β'* and *β* − > *α'*; (d) repeat steps (a)-(c) until a stopping condition is met. Following the work of Strona *et al.*, we use a conservative stopping condition of twice the number of nodes in the network. We perform 10 such randomizations for each network, and store the control profile of each randomization.

### Niche Model

The niche model^26^ first assigns each of *N* species a position on the unit interval via uniform random sampling; the "niche value" of a species *i* is denoted as *v*_*i*_. The prey of species *i* includes all species whose values *v*_*j*_ fall within a species-specific range on the unit interval; the range is centered at or below *v*_*i*_ and has a width drawn from a beta function and scaled by *v*_*i*_ (species with lower positions on the unit interval have correspondingly narrower niches from which they draw prey). The model admits cannibalism and cycles, but maintains the hierarchical trophic structure observed in food webs, along with a number of other properties^26^. The only user-defined parameters in the model are the number of species *N* and the connectance (i.e., density), *C*.

### Configuration Model

To investigate the relationship between the control profile and a network's degree distributions, we applied the configuration model of Newman *et al.*^29^ as implemented in the Python package NetworkX^39^. The configuration model generates a network with a specified joint-degree distribution via random edge assignment. Networks wherein the in-degree distribution and out-degree distributions are preserved but the joint-degree distribution is not may be generated by randomizing the distributions prior to input.

### Fitting the Degree Distribution

The degree distributions were analyzed with the "powerlaw" software package written for Python by Alstott *et al.*^40^ and based on the work of Clauset *et al.*^41^ and Klaus *et al.*^42^. The software applies maximum-likelihood fitting to log-binned degree distributions, among other methods, to accurately determine the distribution's scaling behavior. We compared the data to six candidate distributions: power law, power law with exponential cutoff (i.e., a truncated power law), exponential, stretched exponential, and log-normal.

We report the optimal fit and the *p* values corresponding to the log-likelihood ratio between distributions. In some cases, multiple candidate distributions were valid at *p* > 0.05. A standard example of this is a case where a power law with very weak cutoff (i.e. high maximal degree) fits equally well as a power law with no cutoff.

## Additional Information

**How to cite this article**: Campbell, C. *et al.* Topological constraints on network control profiles. *Sci. Rep.* **5**, 18693; doi: 10.1038/srep18693 (2015).

## Supplementary Material

Supplementary Information
