# Supplementary material for: Topological constraints on network control profiles
Source: Sci Rep. 2015 Dec 22;5:18693. doi: 10.1038/srep18693 (PMC4686937; doi:10.1038/srep18693)
Supplement: Supplementary Information [file srep18693-s1.pdf]

Supplementary Information for

Topological constraints on network control profiles

Colin Campbell, Justin Ruths, Derek Ruths, Katriona Shea, and Réka Albert

Contents:

Supplementary Figures 1 - 7
Supplementary Materials
Supplementary Table 1

Supplementary Figures

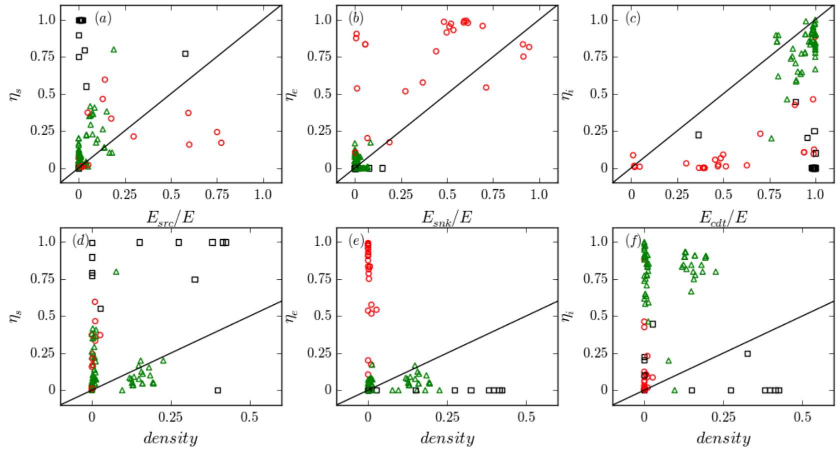

Figure S1. Relationships between the distributions of (a-c) source (E_{src}), sink (E_{snk}) and conduit (E_{cdt}) edges and (d-f) network density and the network control profile parameters among 98 empirical networks. Networks with maximal control profile parameters of η_s, η_e, and η_i are respectively drawn with black squares, red circles, and green triangles. Black lines are drawn through the origin with a slope of 1 as a visual reference. (a) Each source node must be directly controlled, but networks with the largest values of η_s are not necessarily those with the largest relative fraction of edges connected to source nodes. (b) In cases where the number of edges attached to sink nodes is high, η_e is unambiguously the dominant control profile parameter; while sufficient, this is not necessary for a network to have a high value of η_e. (c) Networks with high values of η_i generally have many edges connected to neither source nodes nor sink nodes, but the converse does not hold. The control profile parameters similarly do not scale directly with the network density (d-f).

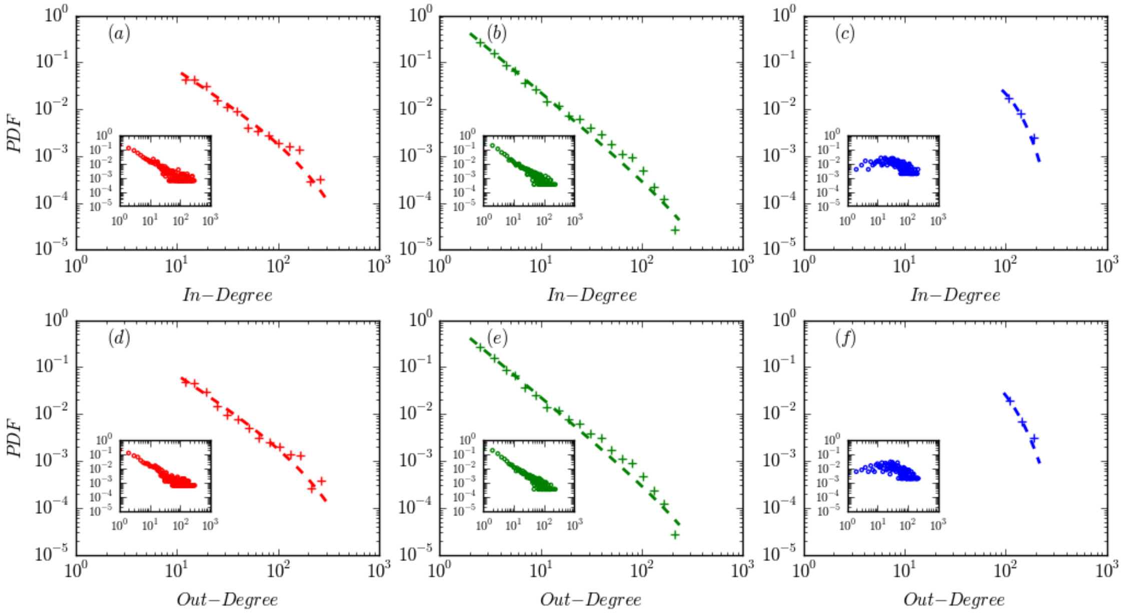

Figure S2. In-degree distributions (a-c) and out- degree distributions (d-f) for the U.S. (red; a,d), international (green; b,e), and 500 busiest (blue; e,f) airport networks. (a-c) Symbols indicate the log-binned empirical probability distribution function over the range of data fitted with best fit curves (dashed lines). Insets show linearly binned distributions. The U.S. and international networks are fit by truncated power laws ($p < 10^{-5}$). While the 500 busiest airports are best-fit by a stretched exponential distribution, the quality of the fit is not significantly greater than a truncated power law ($p > 0.5$; see Methods).

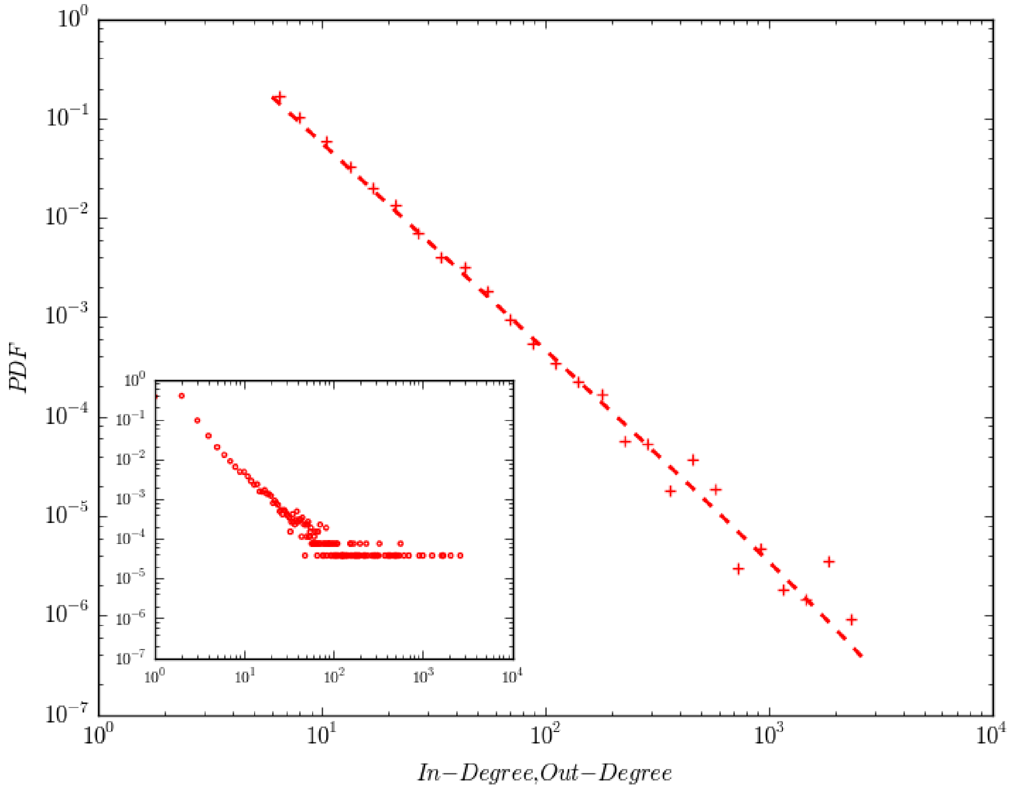

Figure S3. In- and out-degree distributions for the Autonomous System network considered in (13). Symbols indicate the log-binned empirical probability distribution function over the range of data fitted with a best fit curve (dashed line). The inset shows the complete, linearly binned distribution. While we show here a truncated power law, the quality of the fit is not significantly greater than that of a power law or lognormal distribution ($p > 0.2$ in all cases; see Methods).

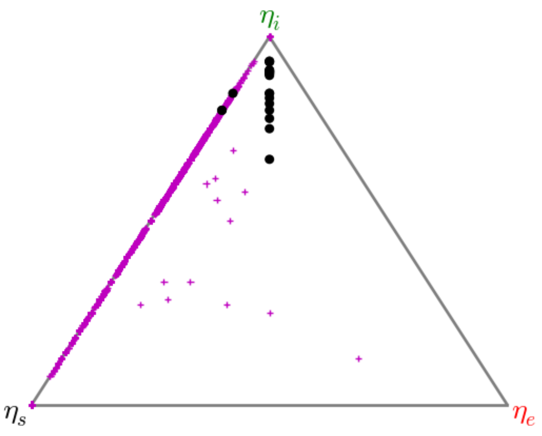

Figure S4. The control profile of empirical (black circles) and simulated (magenta "+" symbols) food webs. For each empirical network, 100 simulated networks are generated with (a) the niche model or (b) with a modified niche model that controls for the number of source nodes.

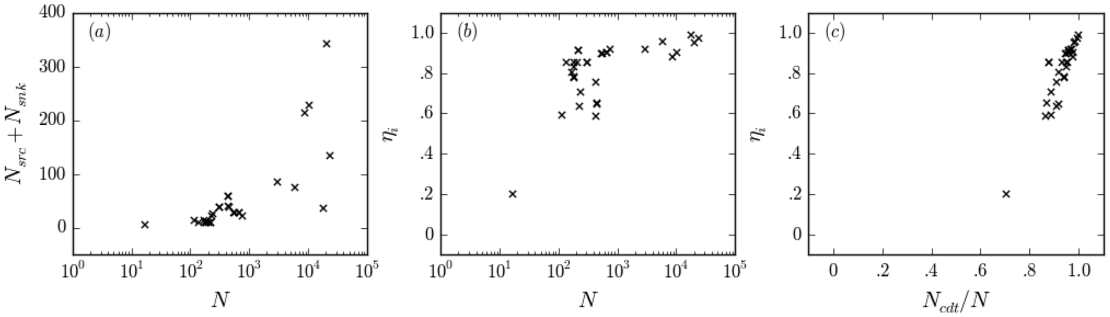

Figure S5. Properties of the 31 ISCAS89 benchmark circuits. (a) The number of source and sink nodes grows slowly compared to the size of the networks. (b) The control profile becomes dominated by η_i for networks with more than 1000 nodes. (c) The value of η_i scales linearly with the fraction of conduit nodes (i.e., those that are neither sources nor sinks).

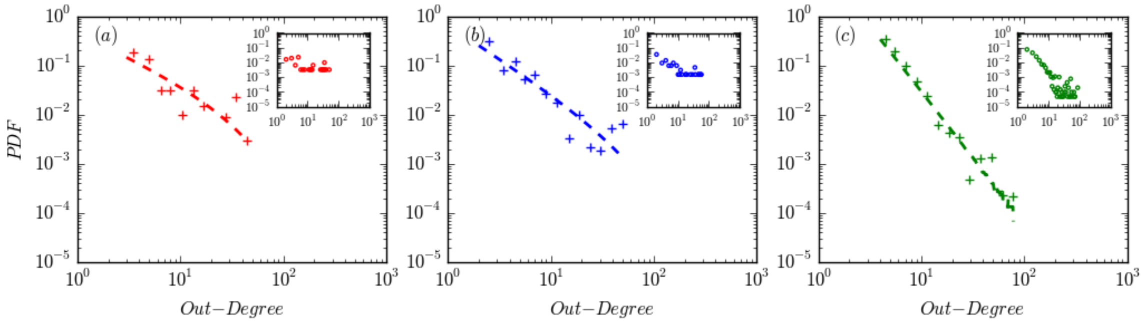

Figure S6. Out degree distributions for the s832 (310 nodes), s1494 (661 nodes), and s38584 (20717 nodes) circuits from the ISCA89 set of benchmark networks. Symbols indicate the log-binned empirical probability distribution function over the range of data fitted by best fit curves (dashed curves). Insets show linearly binned distributions. Panel (a) is fit by a truncated power law, though both exponential and stretched exponential distributions are statistically equivalent ($p > .1$ in both cases; see Methods), while panels (b) and (c) are respectively fit by a truncated power law and a lognormal distribution.

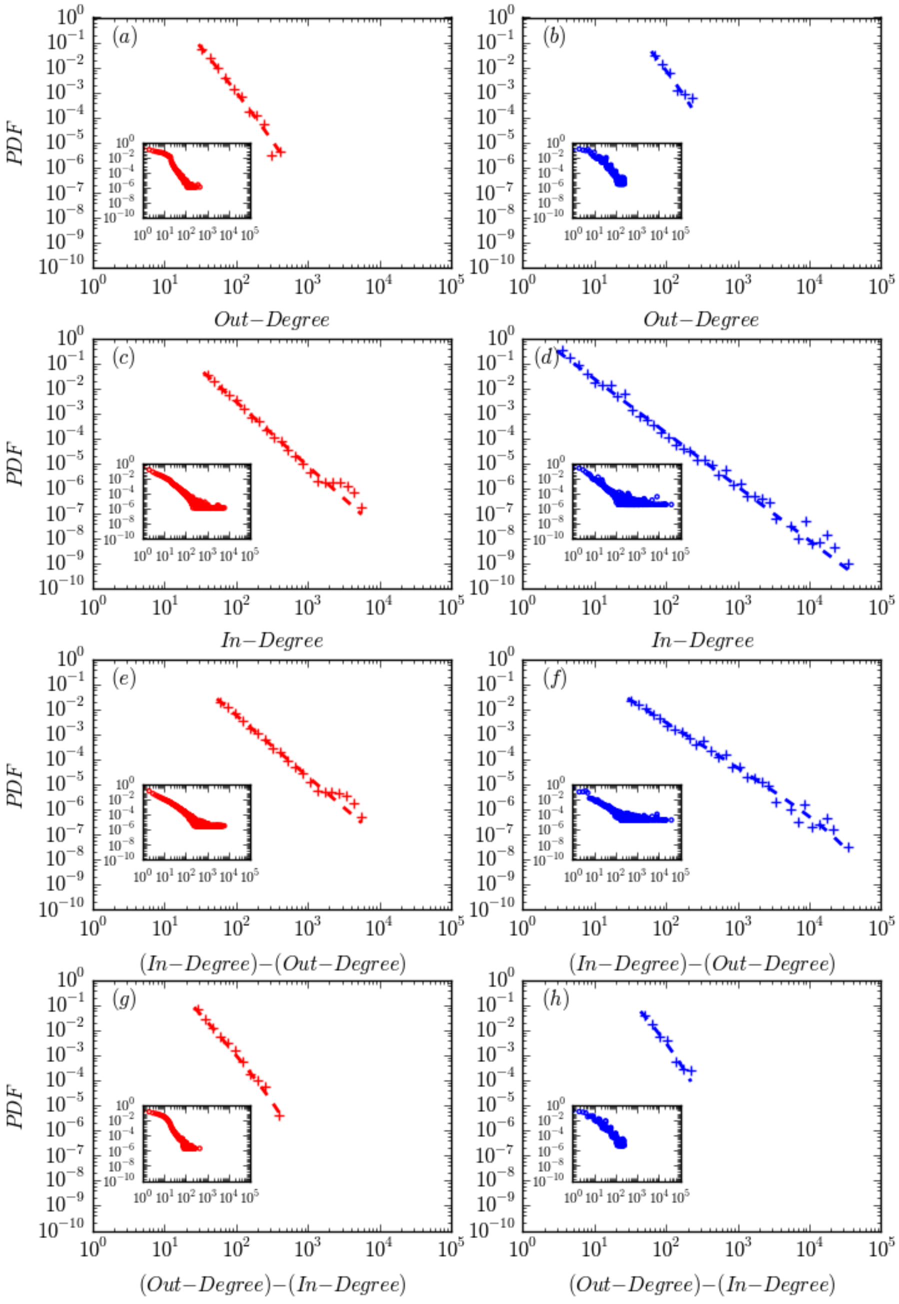

Figure S7. Distributions for the Google (left panels) and Stanford (right panels) samples of the World Wide Web. Panels (e-h) show the difference in in-degree and out-degree only for nodes where in-degree > out-degree; panels (g-h) show the reverse. All distributions are shown with truncated power laws, although power law and log-normal distributions are statistically equivalent in the case of panels (a,c,e) ($p > 0.1$) and log-normal distribution is statistically equivalent in the case of panels (f,h) ($p > 0.09$).

Supplementary Materials

We analyzed 98 empirical networks whose control profiles display dominance by each of the three control profile parameters. Our data set was formed by first selecting the 70 empirical networks analyzed by Ruths and Ruths (13), then replacing the three electronic circuit networks of that data set with a complete representation of the 31 ISCA89 circuits described by Brglez et al. (27).

In Table S1, we enumerate these networks and provide their online locations. A superscript † symbol indicates that the directionality of the network's edges have been reversed for our analysis (see ref. 13).

Table S1. Networks used in this study.

13	***η_s-dominated networks***	
**4**	**neural**	
1	*C. Elegans*	http://toreopsahl.com/datasets
3	Macaque	http://cocomac.g-node.org/
		https://sites.google.com/site/bctnet/datasets
		http://www.biological-networks.org/
**9**	**social**	
1	Email-EU	http://snap.stanford.edu/data/
4	Intra-Organizational†	http://toreopsahl.com/datasets/
1	Physician†	http://moreno.ss.uci.edu/data.html
		friendship network (1 of 3 from this source)
2	Slashdot†	http://snap.stanford.edu/data/
1	Pokec†	http://snap.stanford.edu/data/
24	***η_e-dominated networks***	
**4**	**copurchase**	
4	Amazon	http://snap.stanford.edu/data/
**1**	**corporate ownership**	
1	Corporate ownership	http://vlado.fmf.uni-lj.si/pub/networks/data/econ/Eva/Eva.htm
**2**	**messaging**	
2	UC-Irvine	http://toreopsahl.com/datasets/
**9**	**p2p**	
9	Gnutella	http://snap.stanford.edu/data/
**6**	**social influence**	
2	Physician†	http://moreno.ss.uci.edu/data.html
1	Epinions†	http://snap.stanford.edu/data/
1	Teacher-student	http://moreno.ss.uci.edu/data.html
2	Wikipedia	http://snap.stanford.edu/data
		wiki-Vote edge directionality reversed
**2**	**transcription**	
1	*E. coli*	http://www.weizmann.ac.il/mcb/UriAlon/
1	Yeast	http://www.weizmann.ac.il/mcb/UriAlon/
**61**	**η_e-dominated networks**	
**3**	**airport**	
2	airports	http://toreopsahl.com/datasets/
1	Airports-500	http://www.biological-networks.org/
**1**	**autonomous systems**	
1	autonomous	http://snap.stanford.edu/data/
		final snapshot (Nov. 5, 2007)
**31**	**electronic circuits**	
31	ISCA89	http://www.pld.ttu.ee/~maksim/benchmarks/
**22**	**food webs**	
22	Food webs	http://vlado.fmf.uni-lj.si/pub/networks/data/bio/foodweb/foodweb.htm
**4**	**WWW+blog**	
1	Political blog†	http://www-personal.umich.edu/~mejn/netdata/
3	WWW	http://snap.stanford.edu/data/
		includes berkely.edu, stanford.edu, and google.com
